# The prevalence of unintended pregnancy and its influence on pregnancy experience in Tabriz, Iran, 2023: a cross-sectional study

**DOI:** 10.1186/s12978-024-01821-1

**Published:** 2024-06-05

**Authors:** Mahsa Maghalian, Roghayeh Nikanfar, Mahsan Nabighadim, Mojgan Mirghafourvand

**Affiliations:** 1 Student Research Committee, Midwifery Department, Faculty of Nursing and Midwifery, Tabriz University of Medical Sciences, Tabriz, Iran; 2 Tabriz University of Medical Sciences, Tabriz, Iran; 3 Medical School, Ardabil University of Medical Sciences, Ardabil, Iran; 4 Social Determinants of Health Research Center, Tabriz University of Medical Sciences, Tabriz, Iran

**Keywords:** Unplanned pregnancy, Prevalence, Experience, Predictors, Association, Cross-sectional study

## Abstract

**Background:**

There is a lack of quantitative studies that specifically measure the association between the experience of pregnancy and unintended pregnancy. The present study aims to address the prevalence of unintended pregnancy and identify its predictors. Additionally, the study explores whether unintended pregnancy is associated with pregnancy uplifts and hassles.

**Methods:**

This cross-sectional study was conducted on 488 pregnant women between 20 to 40 weeks' gestation at the comprehensive health center in Tabriz City from February 2022 to January 2023. A cluster sampling method was used for sampling, and data were collected using socio-demographic questionnaires and the Pregnancy Experience Scale (PES). Descriptive statistics were used to describe the socio-demographic characteristics and the prevalence of unintended pregnancy. Binary logistic regression was employed to identify the predictors of pregnancy desirability. To examine the relationship between unintended pregnancy and pregnancy experience, an independent t-test was used for bivariate analysis, and a general linear model (GLM) was utilized for multivariate analysis, with control for potential confounding variables.

**Results:**

The prevalence of unintended pregnancies was 30.7% (24.3% unwanted pregnancies, and 6.4% mistimed pregnancies). The results of the binary logistic regression indicated that the lower age of both the woman and her spouse were significant predictors for unintended pregnancy (*P* < 0.05). Based on an independent t-test, the mean score for uplifts in women with unintended pregnancy was significantly lower than in women with intended pregnancy (mean difference (MD): -4.99; 95% confidence interval (CI): -5.96 to -4.02; *p* < 0.001), While the mean score of hassles in women with unintended pregnancy was significantly higher than women with intended pregnancy (MD: 2.92; 95% CI: 2.03 to 3.80; *p* < 0.001). The results of GLM showed that women who had unintended pregnancies had significantly lower scores for uplifts (B = -4.99; 95% CI: -5.96 to -4.03; *P* < 0.001) and higher scores for hassles (B = 2.92; 95% CI: 2.06 to 3.78; *P* < 0.001).

**Conclusions:**

The high prevalence of unintended pregnancies in Tabriz highlights the importance of targeted interventions to address this issue, considering the policy framework and unique challenges faced by women. Future studies should focus on developing context-specific interventions that effectively meet the needs of women with unintended pregnancies.

## Plain Language Summary

An unintended pregnancy is a pregnancy that occurs either when the woman did not intend to get pregnant at all or when she intended to get pregnant but became pregnant at an inappropriate time. This type of pregnancy can have negative effects on the physical and mental health of women during pregnancy and after delivery. So far, no study has investigated the relationship between the experience of pregnancy and unintended pregnancy, and the existing studies, which are qualitative and based on interviews, have examined women's experiences of unintended pregnancy. For the first time, our study examined the relationship between pregnancy experience and unintended pregnancy in 488 women using a valid questionnaire, the Pregnancy Experience Scale, which includes two parts: uplifts and hassles specific to pregnancy. Our findings showed that women who have an unintended pregnancy have a worse pregnancy experience in both uplifts and hassles specific to pregnancy. Also, limited studies have investigated the prevalence of unintended pregnancy in Iran, especially in the city of Tabriz in recent years. Our study, found this prevalence to be 30.7% in Tabriz, with 24.3% of women having no intention of getting pregnant and 6.4% of women experiencing pregnancy at an inappropriate time. The recommendation is to implement strategies to reduce unintended pregnancy rates and improve women's knowledge of fertility, sexuality, and contraception.

## Background

An unintended pregnancy refers to a situation where a woman either did not intend to conceive at all or intended to conceive but at an inappropriate time [1–3]. Some risk factors associated with unintended pregnancy include the woman's age, wealth index, place of residence, number of children, intention to use contraception, living arrangements (alone or with a partner), age at the start of cohabitation, low socioeconomic status, history of previous abortions, and history of depression [4–7]. Globally, around 44% of pregnancies from 2010 to 2014 were unintended. The rate of unintended pregnancies is significantly higher in developing regions compared to developed ones [1]. In 2012, out of around 213 million pregnancies globally, 89% occurred in developing nations, with over half of these pregnancies being in Asia [2]. It has also been shown that from 2015 to 2019, there were 121 million unintended pregnancies annually, resulting in 64 unintended pregnancies per 1,000 women aged 15–49 [3]. The prevalence of unintended pregnancies in Iran has been reported differently in various studies, ranging from 17% in the city of Shiraz to 27.8% in the city of Arak [8, 9].

Unintended pregnancy, along with the consequent unsafe abortions, can impose significant financial and social burdens. These burdens can affect fertility rates, diminish the quality of life, and strain the public healthcare system [10–12]. Unintended pregnancy poses a challenging situation for women and public health in low- and middle-income countries, and it is associated with adverse maternal and infant outcomes [13]. These women are more at risk of cesarean section and excessive weight gain during pregnancy [14]. Women with unintended pregnancies are less likely to receive adequate prenatal care and are more likely to delay starting prenatal care. They also tend to use folic acid inappropriately and below the recommended amount. Additionally, they may continue to smoke during pregnancy and are less likely to breastfeed for 8 weeks or more [15, 16]. Unintended pregnancy is associated with low birth weight, preterm birth, as well as incomplete infant immunization [17–19]. These women may also experience complications during pregnancy such as preeclampsia, bleeding, and postpartum eclampsia [20].

Furthermore, women may resort to unsafe and illegal interventions, such as unsafe abortions, to terminate unintended pregnancies. These interventions can have detrimental effects on both the fetus and the mother's health, including complications such as peritonitis, uterine rupture, small bowel perforation, and fetal complications [21, 22]. Globally, it has been demonstrated that out of every 1000 women with unintended pregnancies, approximately 39 cases result in abortion [3].

Unintended pregnancy has varying impacts on women, and some individuals may experience more adverse consequences than others. It can lead to a decrease in the quality of relationships with partners and the ability to adapt to the work environment. Personal well-being and a negative outlook on life are mechanisms through which unintended pregnancies can have undesirable effects on a woman's life [23]. In addition, the negative experience of unintended pregnancy can lead to undesirable psychological disorders, including anxiety, post-traumatic stress disorder (PTSD), and postpartum depression [24, 25].

Some qualitative studies have been conducted in Iran [26–29], particularly among the Azari ethnicity [22], to examine women's experiences of unintended pregnancy. These studies consistently demonstrate the negative effects of such pregnancies on the physical and psychological health of mothers, including resorting to abortion as a coping mechanism and experiencing feelings of guilt afterward, fear of judgment from their family, friends, community, or society reactions, and anxiety about taking on the role of motherhood. However, there has been a lack of quantitative studies investigating women's experiences of these pregnancies.

Given the negative impact of unintended pregnancy on health and quality of life, as well as the scarcity of quantitative studies exploring the relationship between pregnancy experience and this particular type of pregnancy, and the limited research on the prevalence of unintended pregnancy in Iran, including the absence of a study conducted in the city of Tabriz that considers both unwanted and mistimed pregnancies, the present study was undertaken with the following objectives:

1. To determine the prevalence of unintended pregnancy.
2. To identify the predictors of unintended pregnancy.
3. To examine whether unintended pregnancy is a predictor of pregnancy uplifts.
4. To investigate whether unintended pregnancy is a predictor of pregnancy hassles.

## Methods

### Study setting and participants

The present study was a cross-sectional study conducted to determine the prevalence of unintended pregnancy and its impact on the pregnancy experience among 488 eligible pregnant women in healthcare centers in Tabriz City from February 2022 to January 2023.

The inclusion criteria for the study consisted of pregnant women between 20 to 40 weeks' gestation, having physical and mental health based on the mother's self-report, the absence of any known diseases or abnormalities in the fetus based on the pregnant woman's medical records, being primiparous or multiparous and having a singleton pregnancy.

The exclusion criteria included women who were unwilling to participate, those who had experienced a significant adverse event in the past three months (such as the death of a family member or divorce), those who had incomplete responses to more than 20% of the questionnaire items, and those with a history of psychiatric illnesses (such as severe depression or requiring medication) based on the mother's self-report and medical records.

### Sample size

The sample size was determined based on the study conducted by Almasi-Hashiani, using the single proportion formula. Considering the prevalence of unintended pregnancy as 19.81%, an alpha (α) value of 0.05, and a study precision of 0.05, the initial sample size was calculated to be 244. However, since the sampling method used was cluster sampling with a design effect of 2, the final sample size was adjusted to 488 individuals [30].

$$\text{N}=\frac{\left(Z_{1-a/2}\right)^2\;\ast p(1-p)}{d^2}$$

### Data collection

After obtaining ethical approval from the Tabriz University of Medical Sciences (IR.TBZMED.REC.1400.681), permission for sampling was granted. Cluster sampling was employed in the study. Initially, one-fourth of the healthcare centers in Tabriz city (21 comprehensive health centers) were randomly selected using the website www.random.org. The researcher then visited the comprehensive health center and extracted a list of pregnant women between 20 and 40 weeks of gestation from the available records (SIB system). From the prepared list, the required number of samples for each center was determined proportionally and randomly selected. Subsequently, contact was made with these individuals via telephone, during which the research objectives and methods were briefly explained. During the phone call, individuals were assessed for inclusion and exclusion criteria, and if eligible and willing to participate in the research, they were invited to attend the designated center at the scheduled time. In the face-to-face meeting, the research objectives and methods were fully explained, and if pregnant women were willing to cooperate, the socio-demographic questionnaire and pregnancy experience scale (PES) were completed through interviews with the participants.

### Data collection tools

This study collected data using the socio-demographic questionnaire, and the Pregnancy Experience Scale (PES).

#### Socio-demographic questionnaire

This questionnaire includes questions about age, level of education, employment status of the woman and her spouse, income sufficiency, and unintended pregnancy (unwanted or mistimed). To determine the validity of this questionnaire, content, and face validity were assessed by the faculty members of the Tabriz University of Medical Sciences.

#### Pregnancy Experience Scale (PES)

The PES, developed by Janet A. DiPietro in 2008, was utilized to assess the pregnancy experience. The questionnaire consists of 20 items, with 10 items dedicated to measuring uplifts specific to pregnancy and 10 items focused on hassles specific to pregnancy. Participants provided their responses on a four-point Likert scale, ranging from "Not at all" (score of zero) to "Very much" (score of three) for each item [31]. The validity and reliability of the Persian version of the pregnancy experience questionnaire were assessed in a study conducted by Ebadi et al. [32]. The overall tool demonstrated a reliability coefficient 0.714, indicating satisfactory internal consistency. Moreover, reliability coefficients of 0.777 and 0.672 were reported for the uplifts and hassles subscales, respectively, providing additional evidence of the questionnaire's reliability in measuring specific aspects of pregnancy experiences. The Cronbach's alpha coefficients calculated for the current study were 0.70 for the overall instrument, 0.82 for uplifts, and 0.73 for hassles specific to pregnancy. The Cronbach's alpha values of the original scale obtained for the uplifts and hassles subscales were 0.82 and 0.83, respectively.

### Data analysis

After collecting information from all the research participants, the data were analyzed using SPSS-Version 24 software. Descriptive statistics, including frequency (percentage) and mean (SD = standard deviation), were used to describe the socio-demographic characteristics and prevalence of unintended pregnancy. Binary logistic regression was used to identify the predictors of pregnancy desirability. Variables with *p* < 0.05 in the socio-demographic characteristics, as presented in Table 1, were included as independent variables, while pregnancy desirability was designated as the dependent variable in the binary logistic regression analysis. To examine the relationship between unintended pregnancy and pregnancy experience, an independent t-test was employed for bivariate analysis, and a general linear model (GLM) was utilized for multivariate analysis, with control of potential confounding variables.

**Table 1:** Socio-demographic characteristics and their relationship with pregnancy desirability and pregnancy experience

Variables	Relationship with pregnancy desirability			Relationship with pregnancy experience			
	**Intended pregnancy (*n* = 338)**	**Unintended pregnancy** **(*n* = 150)**		**Uplifts**		**Hassles**	
	**N (%)**	**N (%)**	***P* value**	**Mean (SD)**	***P* value**	**Mean (SD)**	***P* value**
**Women’s age** (Years)							
≥ 19	32 (21.3)	36 (10.7)	**0.001**^a^	16.92 ± 5.33	**0.018**^c^	14.32 ± 4.94	0.076^c^
20–29	50 (33.3)	164 (48.5)		18.91 ± 5.16		15.28 ± 4.59	
30–39	58 (38.7)	129 (38.2)		17.42 ± 5.80		14.14 ± 4.58	
≥ 40	10 (6.7)	9 (2.7)		18.0 ± 5.54		15.66 ± 4.06	
**Husband’s age** (Years)							
19–29	29 (19.3)	110 (32.5)	**0.003**^a^	18.48 ± 5.25	0.323^c^	14.02 ± 4.56	0.489^c^
30–39	84 (56)	176 (52.1)		18.0 ± 5.58		15.13 ± 4.54	
≥ 40	37 (24.7)	52 (15.4)		17.35 ± 5.83		14.84 ± 4.95	
**Women’s educational level**							
Primary school	109 (32.2)	38 (25.3)	0.260^a^	18.38 ± 5.60	0.503^c^	15.14 ± 4.54	**0.026**^c^
Secondary school	76 (22.5)	46 (30.7)		17.40 ± 5.28		14.62 ± 4.65	
High school	66 (19.5)	29 (19.3)		18.22 ± 5.58		15.53 ± 4.66	
Diploma	44 (13.0)	22 (14.7)		17.56 ± 5.80		14.46 ± 4.45	
University	43 (12.7)	15 (10.0)		18.60 ± 5.54		13.15 ± 4.75	
**Husband’s education level**							
Primary school	37 (10.9)	13 (8.70)	0.476^a^	16.36 ± 5.06	**0.034**^c^	14.22 ± 4.65	0.541^c^
Secondary school	55 (16.3)	21 (14.0)		17.51 ± 5.98		14.53 ± 4.42	
High school	48 (14.2)	19 (12.7)		17.16 ± 5.24		15.34 ± 5.22	
Diploma	100 (29.6)	57 (38.0)		18.67 ± 5.30		15.05 ± 4.34	
University	98 (29.0)	40 (26.7)		18.58 ± 5.72		14.46 ± 4.79	
**Women’s Job**							
Housewife	292 (86.4)	132 (88.0)	0.666^b^	17.95 ± 5.54	0.439^d^	14.88 ± 4.53	0.175^d^
Employed	46 (13.6)	18 (12.0)		18.53 ± 5.545		13.93 ± 5.26	
**Husband’s job**							
Worker	97 (28.7)	39 (26.0)	0.306^b^	17.33 ± 5.36	0.268^c^	14.64 ± 4.51	0.289^c^
Employee	63 (18.6)	20 (13.3)		18.81 ± 6.12		14.15 ± 4.80	
Shopkeeper	18 (5.30)	7 (4.70)		18.08 (4.41)		13.92 (4.46)	
Self-employment	160 (47.3)	84 (56.0)		18.13 (5.52)		15.11 (4.66)	
**Sufficiency of family income**							
Insufficient	18 (5.30)	13 (8.70)	0.390^a^	18.44 (5.57)	0.467^c^	14.83 (4.66)	0.826^c^
Relatively sufficient	263 (77.8)	113 (75.3)		18.02 (5.49)		14.70 (4.66)	
Completely sufficient	57 (16.9)	24 (16.0)		17.0 (6.02)		15.22 (4.54)	

*SD* Standard Deviation, *N* Number

^a^Chi-square tests

^b^Fisher's exact tests

^c^One-way ANOVA

^d^Independent t-test

## Results

The prevalence of unintended pregnancies in Tabriz-Iran was 30.7%. Out of this percentage, 24.3% were identified as unwanted pregnancies, while 6.4% were mistimed pregnancies.

There was no significant difference in socio-demographic characteristics among participants with unintended and intended pregnancies except of age of the woman (*p* = 0.001) and age of the husband (*p* = 0.003) (Table 1).

The results of the binary logistic regression analysis indicated that the lower age of both the woman and her spouse were significant predictors for unintended pregnancy. Women aged under 19 years (adjusted odds ratio [aOR]: 2.91; 95% CI: 1.64–5.16; *p* < 0.001) and those aged 20–29 years (aOR: 1.97; 95% CI: 1.12–3.48; *p* = 0.019), as well as their spouses aged 19–29 years (aOR: 2.69; 95% CI: 1.50–4.85; *p* = 0.001), had a higher chance of experiencing unintended pregnancies compared to higher ages (Table 2).

**Table 2:** Factors associated with pregnancy desirability based on binary logistic regression

**Variable**	**OR**	**(95%CI**^a^**)**	***P* value**
**Women’s age**			
≥ 19	2.91	1.64–5.16	** < 0.001**
20–29	1.97	1.12–3.48	0.019
30–39	0.80	2.89–2.21	0.668
≤ 40 (Reference)	1		
**Husband’s age**			
19–29	2.69	1.50–4.85	**0.001**
30–39	1.49	0.90–2.44	0.114
≤ 40 (Reference)	1		

^a^Confidence interval

Among women with unintended pregnancy, the mean ± SD of uplifts was 14.56 ± 5.53, and the mean ± SD of hassles was 16.78 ± 4.69 (obtainable score range of 0 to 30). In contrast, among women with intended pregnancy, the mean ± SD of uplifts was 19.56 ± 4.80, and the mean ± SD of hassles was 13.86 ± 4.33, within the same score range of 0 to 30. Based on an independent t-test, the mean score of uplifts in women with unintended pregnancy was significantly lower than in women with intended pregnancy (mean difference (MD): -4.99; 95% confidence interval (CI): -5.96 to -4.02; *p* < 0.001), While the mean score of hassles in women with unintended pregnancy was significantly higher than women with intended pregnancy (MD: 2.92; 95% CI: 2.03 to 3.80; *p* < 0.001) (Table 3).

**Table 3:** Comparison of pregnancy experience in participants with unintended or intended pregnancy

Pregnancy experience	**Intended pregnancy** ***N* = 338**	**Unintended pregnancy** ***N* = 150**	**Mean difference (95%CI)**	**Obtained score range**	**Obtainable score range**	***P* value**^a^
	**Mean** ± **SD**	**Mean** ± **SD**				
**Uplifts**	19.56 ± 4.80	14.56 ± 5.53	-4.99 (-5.96 to -4.02)	4–30	0–30	< 0.001
**Hassles**	13.86 ± 4.33	16.78 ± 4.69	2.92 (2.03 to 3.80)	3–28	0–30	< 0.001

*CI* confidence interval

^a^Independent t-test

Based on bivariate analysis, the variables of women's age (*p* = 0.018) and husband's education level (*p* = 0.034) showed a relationship with uplifts. Additionally, women's educational level (*p* = 0.026) and women's jobs (*p* = 0.175) were found to be related to hassles, with a significance level of *P* < 0.2. These variables, along with the unintended pregnancy variable, were included as independent variables in the GLM, with hassles and uplifts serving as the dependent variables.

The findings indicated that women who experienced unintended pregnancies exhibited significantly lower scores for uplifts (B = -4.99; 95% CI: -5.96 to -4.03; *P* < 0.001) and higher scores for hassles (B = 2.92; 95% CI: 2.06 to 3.78; *P* < 0.001) compared to women with intended pregnancies. Additionally, women with education below diploma level, specifically primary school (B = 1.98; 95% CI: 0.58 to 3.39; *P* = 0.006), secondary school (B = 1.46; 95% CI: 0.02 to 2.91; *P* = 0.046), and high school (B = 2.38; 95% CI: 0.87 to 3.89; *P* = 0.002) also obtained higher scores for hassles compared to those with university education (Table 4).

**Table 4:** Relationship of unintended pregnancy with pregnancy experience based on adjusted general linear model

Variable	B (95% CI^a^)	*P*-value
**Pregnancy experience (Uplifts)**		
**Unintended pregnancy**		
Yes	-4.99 (-5.96 to -4.03)	** < 0.001**
No	0	
**Husband’s education level**		
Primary school	0.37 (-1.42 to 2.17)	0.685
Secondary school	0.30 (-1.24 to 1.86)	0.697
High school	0.81 (-0.80 to 2.44)	0.323
Diploma	-0.31 (-1.58 to 0.96)	0.633
University	0	
**Women’s age**		
≥ 19	-0.79 (-3.60 to 2.00)	0.577
20–29	1.16 (-1.42 to 3.75)	0.377
30–39	-0.28 (-2.89 to 2.31)	0.828
≥ 40	0	
**Pregnancy experience (Hassles)**		
**Unintended pregnancy**		
Yes	2.92 (2.06 to 3.78)	** < 0.001**
No	0	
**Women’s educational level**		
Primary school	1.98 (0.58 to 3.39)	**0.006**
Secondary school	1.46 (0.023 to 2.91)	**0.046**
High school	2.38 (0.87 to 3.89)	**0.002**
Diploma	1.31 (-0.31 to 2.94)	0.114
University	0	
**Women’s Job**		
Housewife	0.94 (-0.27 to 2.17)	0.127
Employed	0	

^a^Confidence interval

## Discussion

The results of the present study showed that approximately one-third of women in the city of Tabriz had unintended pregnancies. Furthermore, unintended pregnancy was identified as a predictive factor for a worse pregnancy experience.

In the current study, the prevalence of unintended pregnancy was found to be 30.7%, which is higher than the reported prevalence in countries such as Saudi Arabia (26.4%) and Ethiopia (28%) [33, 34]. Moreover, it is higher than the prevalence of unintended pregnancy reported in the latest study conducted in Iran, where in a cross-sectional study of 5152 pregnancies in 103 hospitals in Tehran (the capital city) in 2015, it was considered to be 19.81% [30]. Furthermore, in a cross-sectional study conducted in 2014 in Kermanshah, West Iran, involving 248 women aged 15–49, the prevalence of unintended pregnancy was estimated to be 2.21% [35]. Additionally, in a survey conducted in 2013 in Shiraz City, the prevalence of unintended pregnancy was estimated to be 17% [4]. In another analytical cross-sectional study of 352 mothers with infants aged 6–12 months in 2007 in Arak City, the prevalence of unintended pregnancy was 8.27% [5]. This difference could be attributed to population growth policies in Iran in recent years and the fact that this study was conducted during the COVID-19 pandemic. In a cross-sectional study conducted in Iran to compare contraceptive methods, abortion, and unintended pregnancies before and during the COVID-19 pandemic, it was found that the prevalence of unintended pregnancies increased from 20.4% before the pandemic to 25.4% during the pandemic [36].

According to a study [37], it has been found that women's reports of pregnancy planning during the postpartum period can lead to unreliable information. In a cross-sectional study conducted in Tabriz in 2020 by Taheri et al. [38], they reported a prevalence of 26.2% for unwanted pregnancy, while our current study reports a slightly lower prevalence of 24.3%. These differences in findings may be attributed to the evaluation of pregnancy planning during both the pregnancy and postpartum periods, as well as the inclusion of participants from diverse ethnic backgrounds in Taheri et al.'s study. In our present study, we specifically examined unwanted pregnancy during pregnancy among Azeri women.

Significant differences were observed in terms of uplifts and hassles between the two groups of pregnant women classified based on their intended and unintended pregnancies. Specifically, the group with unintended pregnancies demonstrated lower levels of uplifts and higher levels of hassles. However, it is important to note that there is currently a lack of quantitative research investigating this aspect. Several qualitative studies have demonstrated the negative effects of unintended pregnancy on women's pregnancy experience [22, 26, 27, 28, 29, 39, 40]. In a qualitative study conducted in Tabriz, Iran, Mohammadi et al. demonstrated that unintended pregnancy poses challenges for women and can result in social deprivation, physical and psychological risks, as well as unsafe and illegal consequences of abortion for both the mother and the infant [22]. Furthermore, several other qualitative studies conducted in Iran have also revealed that unintended pregnancy creates significant psychological pressures for women. Factors such as unpreparedness, both physically and emotionally, financial constraints in accepting the pregnancy, adherence to the two-child policy, feelings of embarrassment regarding pregnancy in the presence of other children and relatives, conflicting religious beliefs, and contemplation of fetal abortion are among the perceptions associated with unintended pregnancy among women [26–29]. Additionally, it should be noted that unintended pregnancy and low educational attainment in women can lead to psychological consequences, which, in turn, can impact the overall pregnancy experience [41].

Results showed that unintended pregnancy can serve as a predictor of both uplifts and hassles. The emotional impact of unintended pregnancy on women is significant, as it often leads to a sense of ambivalence and uncertainty. Furthermore, women experiencing unintended pregnancies tend to report lower levels of happiness [42]. The absence of prior pregnancy planning and the sudden shift in circumstances can contribute to heightened levels of anxiety among women [43]. However, it is important to note that personal resilience and individual circumstances can also influence the emotional outcomes of unplanned pregnancies [44].

The findings of the present study also highlight a significant positive correlation between women's educational level and the experience of hassles. Specifically, our results indicate that women with below high school education tend to report higher hassle scores. This observation aligns with previous research indicating that women with lower levels of education are more susceptible to adverse pregnancy outcomes, such as stillbirth [45]. Moreover, educational attainment has been found to be associated with fertility and sexual knowledge, which in turn can influence the overall pregnancy experience [46, 47]. It is worth noting that attitudes towards fertility, education, and awareness play a crucial role in predicting the inclination towards future pregnancies, particularly in the context of the first and second child [48].

In the present study, we also found that a lower age of both the woman and her spouse was identified as a risk factor for unintended pregnancy. These findings are consistent with previous studies [4, 5, 49], which have similarly shown that a lower age is a predictor of unintended pregnancy. This observation may be attributed to limited access to high-quality sexual and reproductive health information among individuals in these age groups [49]. Limited access to education and family planning services presents a particular challenge for young and healthy women due to Iran's population growth policies [4, 23]. Additionally, the flexibility of the work environment for employed women is another influential factor in their experience of unintended pregnancy [23]. In response to these challenges, Iran has implemented new population policies that include strategic measures to support young and healthy women, such as providing postpartum leave with wages and the option to work from home. These provisions aim to facilitate a better balance between family and work life [50].

Furthermore, contrary to previous studies [51, 52], although the educational level of the woman was not identified as a risk factor for unintended pregnancy in the present study, lower education level of women was found to be a predictor of a worse pregnancy experience among women with unintended pregnancies.

It is necessary to consider interventional studies in light of Iran's population increase policy. These interventions include promoting the continuation of pregnancies, preventing abortions, educating women about the mental and physical risks associated with abortion, and facilitating a positive childbirth experience. It is also important to provide counseling services to women and their families, as well as educate and encourage them about the importance of parenthood and having children to strengthen families. Furthermore, it would be essential to quantitatively evaluate the impact of such interventions on the pregnancy experience. Additionally, we recommend conducting mixed-method studies to obtain comprehensive data on unintended pregnancy experiences while considering the proposed interventions.

### Strengths and limitations

The strengths of the current study include the quantitative investigation of the relationship between pregnancy experience and unintended pregnancy for the first time. This study also utilized standardized tools to measure the pregnancy experience. Additionally, the study employed a random selection method to choose participants from healthcare centers in Tabriz city.

The current study has several limitations. Firstly, as a cross-sectional study, it cannot establish a causal relationship between pregnancy experience and unintended pregnancy. Future research using longitudinal designs is needed to address this limitation. Additionally, the generalizability of the findings may be limited due to the focus on healthy singleton pregnancies and a specific ethnic group (Azari ethnicity), which restricts the representation of diverse populations. Furthermore, the study did not include women with pregnancies below 20 weeks' gestational age, multiple pregnancies, women with mental disorders and related medication use, or those who recently experienced significant adverse events. Future research should aim to incorporate these subgroups to ensure a more comprehensive understanding of the experiences of unintended pregnancy.

## Conclusions

The study findings indicated a significantly high prevalence of unintended pregnancies among women in the city of Tabriz. The high prevalence of unintended pregnancies, along with the observed differences in the experiences of uplifts and hassles, highlights the pressing need for targeted interventions. Future studies should take into account the broader policy framework, such as Iran's population increase policy, and address the unique challenges faced by women with unintended pregnancies. This approach will enable the development of context-specific interventions that effectively meet the needs of this specific population.

## Data Availability

Upon request, the corresponding author will provide access to the data.

## Abbreviations

AOR: Adjusted odds ratio
CI: Confidence interval
GLM: General linear model
MD: Mean difference
PES: Pregnancy experience scale
SD: Standard deviation
